# Early life malnutrition and risk of T2DM adulthood: evidence from the lower socioeconomic status of northwest Chinese population

**DOI:** 10.3389/fnut.2024.1379725

**Published:** 2024-06-27

**Authors:** Hongjuan Shi, Danyu Yang, Ling Ma, Yin Cheng, Yining Liu, Jinyu Ma, Huitian Tong, Chao Shi

**Affiliations:** ^1^School of Public Health, Ningxia Medical University, Yinchuan, China; ^2^People's Hospital of Ningxia Hui Autonomous Region, Ningxia Medical University, Yinchuan, China; ^3^Ningxia Clinical Research Institute, People’s Hospital of Ningxia Hui Autonomous Region, Yinchuan, China

**Keywords:** early life malnutrition, socioeconomic status, diabetes, adults, Chinese great famine

## Abstract

**Objective:**

This study aimed to explore whether famine exposure during early life are associated with a high risk of Type 2 Diabetes Mellitus (T2DM) in adulthood and the role of socioeconomic status (SES) on this effect.

**Materials and methods:**

We conducted a secondary data analysis based on data from a cross-sectional survey, collected 3,355 participants born between January 1, 1941 and December 31, 1966. Participants were categorized into four groups based on their date of birth, unexposed (individuals born in 1963–1966), infant exposed (individuals born in 1959–1962), childhood exposed (individuals born in 1949–1958), and adolescent exposed (born in 1941–1948). The association of famine exposure with T2DM risk in adults and conducted separately in plain area and mountain area was assessed using logistics regression model.

**Result:**

22.35% of participants were diagnosed with T2DM, of which 43.47% were from the childhood famine-exposed group, representing the highest proportion among all subgroups (*p* < 0.001). Participants exposed to famine during childhood and adolescence from the lower SES mountain areas showed a significantly higher prevalence of T2DM in adulthood than those from the plain areas (*p* < 0.001). The adolescence stage exposed famine will increase the risk of T2DM in the mountain area (OR 2.46, 95% CI 1.61, 3.77).

**Conclusion:**

No strong evidence demonstrates that exposure to famine during the early life stage increases the risk of developing T2DM in adulthood. However, populations with lower SES are likely to be exposed to more risk factors for T2DM.

## Introduction

Ranking as the eighth leading cause of combined death and disability worldwide, diabetes has risen to prominence as one of the major public health challenges impacting countries globally, including China (1, 2). The International Diabetes Federation (IDF) indicates that 537 million people worldwide was living with diabetes in 2021, and is estimated to increase to 1.31 billion in 2050 (3). This high and increasing prevalence has placed a substantial burden on population and healthcare systems, particularly in areas with low socioeconomic status (SES) and limited healthcare resources. Exploring the risk factors of diabetes is of great significance for prevention and management in these areas.

Type 2 Diabetes Mellitus (T2DM) is the most common form of diabetes that characterized by an elevated blood glucose concentration associated with body’s impaired response to insulin action, and influences individuals of all demographics. Identified risk factors for the development of suboptimal glycaemic control include genetic susceptibility, obesity, sedentary lifestyles, and suboptimal dietary patterns (4, 5). The developmental origins of health and disease (DOHaD) hypothesis suggested that the suboptimal glycaemic control is also associated with environmental exposures during early life (6). In details, nutritional status in the early developmental periods represents a key determinant of long-term health outcomes later in life (7). Sustained exposure to food shortages and undernutrition during this period can increase the risk of suboptimal health conditions in adulthood (8, 9). Previous studies reported significant difference in nutrition between lower and higher SES. The food choices in lower SES areas are likely to be limited, which resulting in residents in these areas tend to select food with higher energy, and also have limited selections for fruit and vegetables (10). This could lead to a dietary pattern with a level of essential nutrients such as vitamin and micro element in the lower SES areas (11).

Famine subjects a significant proportion of a country or region’s population to severe food shortages (12). During the period of 1959–1961, China suffered one of the most severe famines in the world, which was known as “the Great Chinese Famine” (13). Almost all people over 60 years old living in mainland China were impacted, with the total death toll from this famine estimated to be 15–30 million (14, 15). The impact of early-life malnutrition on the risk of T2DM in adulthood has been reported in various countries or regions (7, 16). However, the findings have been inconsistent, whether early malnutrition increases the risk of diabetes in adulthood remains unclear (13, 17).

Among all regions in China, the Northwest area was reported to have inferior SES than the East. For example, the southern regions of Ningxia Hui Autonomous Region (NHAR), the smallest northwestern province of China, were classified as the least accommodating places for humans to live by the United Nations before 1990s (18). The people living in this region not only experienced famine, but also be perceived as at a lower SES for a long time until the beginning of this century. Although the northern portion of NHAR showed a higher socioeconomic status (SES) with a relatively mild degree of severe famine (19) than its southern portion, NHAR as a whole continues to exhibit a lower SES when compared to other areas throughout China.

To the best of our knowledge, no research has thoroughly examined the risk of T2DM in populations from such areas that have historically experienced both severe famine and prolonged poverty. Our study aimed to explore whether (1) famine exposure during infant, childhood and adolescence are associated with a high risk of Type 2 Diabetes Mellitus (T2DM) in adulthood and the role of socioeconomic status (SES) on this effect in such underdeveloped areas compared with an age-balanced control. This research would help understand the health determinants of chronic diseases in vulnerable populations, and guide the development of public health interventions for regions under similar circumstances globally. This would further contribute to reducing health disparities across diverse populations and promoting more equitable health outcomes.

## Materials and methods

### Study design and data source

**Figure 1:**
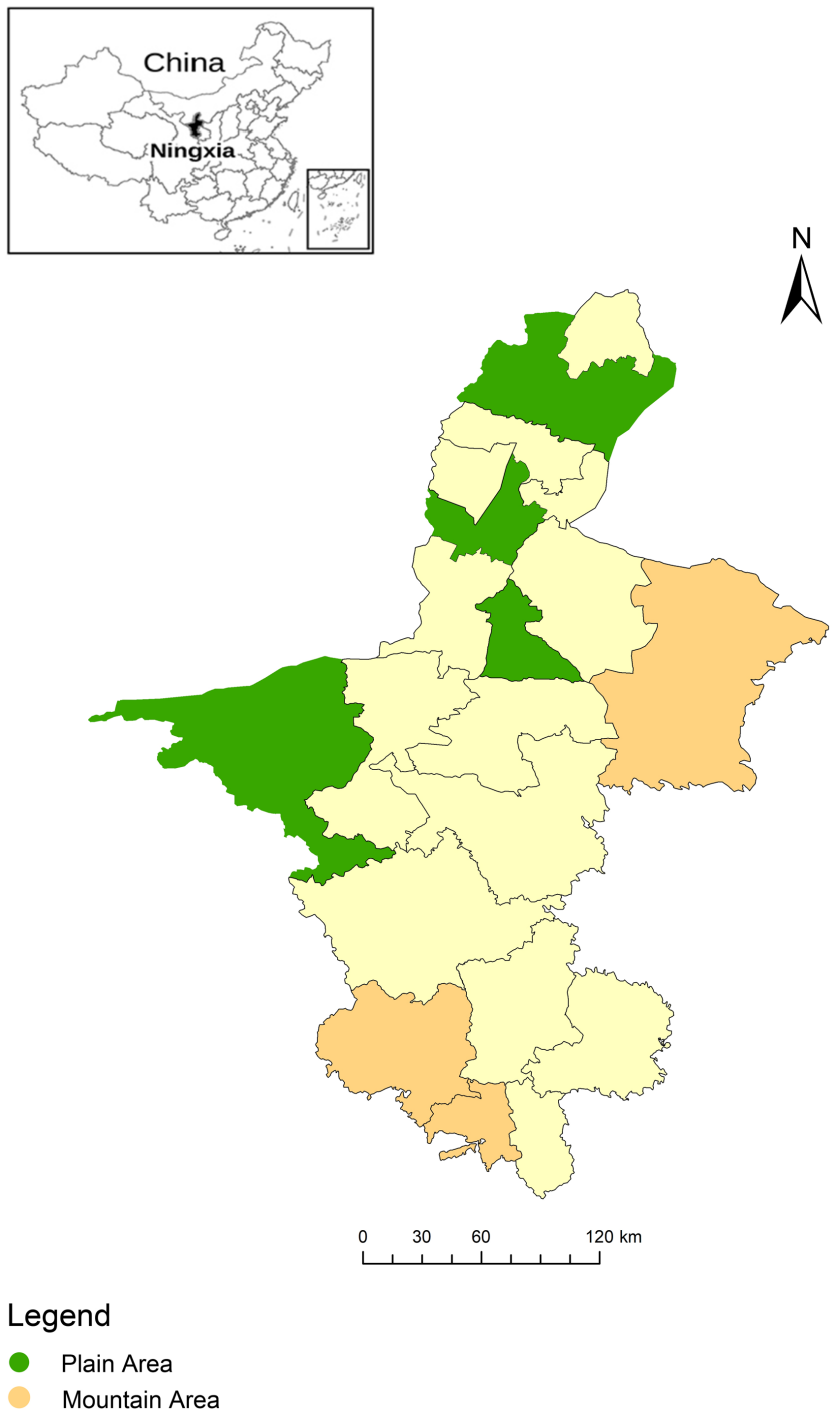
Distribution of study area for cardiovascular disorders and the related risk factors survey in Ningxia Hui Autonomous Region, 2020–2021.

Cross-sectional data from the Ningxia Cardiovascular Disorders Study (NCDS) survey were used. This field-based survey was carried out in NHAR between January 2020 and December 2021 to investigate the prevalence and risk factors of cardiovascular disease including coronary heart disease, obesity, hypertension, dyslipidemia, diabetes mellitus, and hyperuricemia for cardiovascular disease (CVD). Regionally representative samples were selected using a four-stage stratified cluster sampling approach from the general population aged 18 and older. In the first stage, nine counties in NHAR were selected based on economic and administrative levels (Figure 1). In the second stage, two towns from each county were chosen using Simple Random Sampling (SRS) based on the town list provided by the local Centre for Disease Control and Prevention. In the third stage, three communities or villages were selected from each town using SRS. During the final stage, sampling stratification was performed based on age and gender distribution, in accordance with the China census data from 2010.

A total of 10,803 permanent residents were recruited into the cohort with valid baseline data, comprising a face to face questionnaire, physical measurements. Of whom 7,448 were excluded from the present study because of participants who were born not in 1959–1966 & 1941–1948 year, and participants with missing information on the included covariates. Finally, 3,355 individuals were included in the current analysis. The flow chart of the current study design is shown in Supplementary Figure S1.

### Ethical approval

This study was conducted according to the guidelines laid down in the Declaration of Helsinki and all procedures involving human subjects were approved by the Institutional Review Board of the People’s Hospital of Ningxia Hui Autonomous (No. 2020-YC-002). Verbal consent was witnessed and formally recorded. Informed consent was confirmed by the IRB the People’s Hospital of Ningxia Hui Autonomous.

### Participants, exposure age, and area categories

The Great Chinese Famine took place from 1959 to 1961. We conducted a secondary data analysis based on 10,803, extracted 3,355 participants born between January 1, 1941 and December 31, 1966. These individuals were selected as a sample because they were born or in their developmental stages in China during the time that coincided with the Great Chinese Famine, meaning they were highly likely to experience the famine as infants, children or adolescents. Referring to the previous Chinese famine studies (9, 14, 20), These sample participants were categorized into four groups based on their birth dates and the period of famine exposure: participants who were born between January 1, 1959 and December 31, 1962 as the infant exposed group (*n* = 490); participants who were born between January 1, 1949 and December 31, 1958 as the childhood exposed group (*n* = 1,322); participants who were born between January 1, 1941 and December 31, 1948 as the adolescence exposed group (*n* = 767); participants who were born between January 1, 1963 and December 31, 1966 as the non-exposed group (*n* = 776); and non-exposed group and adolescence exposed group were combined as age-balanced control group (*n* = 1,543), this was to control and reduce the bias related to age differences between famine exposure subgroups.

For the study areas, nine counties in NHAR were selected for participation (Figure 1). Among these areas, Xiji County, Longde County and Yanchi County are categorized into the southern mountainous areas, while Jinfeng County, Yongning County, Litong County, Pingluo County, Dawukou County, and Shapotou County are categorized into the northern plain areas.

### Anthropometric data and questionnaires collection

Before anthropometric data collection, all anthropometric information and questionnaires were conducted by healthcare professionals and investigators trained using the same framework. All participants were required to conduct an overnight fasting for 8 hours before the physical assessment. Anthropometric information included waist circumference, weight and standing height were measured by using calibrated instruments under a standardized procedure. When measuring height and weight, the participants being measured was required to be barefoot. Participants’ standing height was measured by a stadiometer with an accuracy of 1 mm under a standardize procedure. After a five-minute rest, the sitting blood pressure of the right arm was measured three times with an electronic blood pressure monitor (OMRON, HBP-1120 U), and the averaged results were collected. Information regarding lifestyle, personal characteristics, health status and SES was collected by well-trained interviewers using a computer-assisted, one-on-one questionnaire.

### Biochemical data measurements

Before biochemical data measurements, medical examinations were performed by trained using the same framework. Fasting blood samples from participants (~5 mL/participant) were collected into heparin sodium-containing anticoagulant tubes and then centrifuged for 10 min at 1500 rpm. The supernatants were then harvested into cryogenic vials, preserved at −80°C and sent to the Beijing CIC Medical Laboratory for analysis with packaged in dry ice. The tests included low-density lipoprotein cholesterol (LDL-C), fasting plasma glucose (FPG), serum uric acid (SUA), triglycerides (TG), serum creatinine (SCr), high-density lipoprotein cholesterol (HDL-C), and total cholesterol (TC). These analyses were performed using a Beckman Coulter AU 5800 device (Beckman Coulter, United States) and commercially available reagents (Biosino, Beijing, China). Glycated hemoglobin (HbA1c) was analysed with the Tosoh Automated Glycohemoglobin Analyzer HLC-723GX (Tosoh Corporation, Japan). All experiments were conducted in accordance with relevant guidelines and regulations.

### Quality control

To minimize variation in implementation of procedures by various staff members over time, standardized operation protocols (SOPs) have been developed, and personnel were trained and certified according to these SOPs. In particular, a quality-control team composed of 18 postgraduates majoring in clinical medicine and epidemiology has been built, two of whom were responsible for a study site. Still more, 5% of participants in each study site were randomly selected and routinely interviewed by quality-control staff to evaluate protocol adherence of the field investigators. In addition, a custom designed epidemiological data collection platform was developed to facilitate data collection, data management, and quality control. It was automatically conducted by the system itself according to the variable value range or required items set in the form.

### Definition of factors including T2DM

T2DM was defined as having a self-reported history of T2DM or being under relevant treatment, a FBG level of ≥7.0 mmol/L, or a HbA1c level of ≥6.5% (21). Body Mass Index (BMI) was defined according to the World Health Organization (WHO) as the weight of an individual in kilograms divided by the square of their height in meters (kg/m^2^). In the current study, BMI was used to categorize individuals as underweight (<24.0 kg/m^2^), within healthy weight (24.0–27.9 kg/m^2^), or overweight (>28.0 kg/m^2^).

### Statistical analysis

All statistical analyses were performed using STATA MP17 (Stata Corp, United States) and the R statistical software packages (http://www.R-project.org, R Foundation, version 4.4.0). All selected demographic and clinical laboratory data were subjected to descriptive statistical analysis. Categorical variables were represented as percentages, while continuous variables with a normal distribution were expressed as means±standard deviation (SD). Pearson’s Chi-Square test was used to identify differences in categorical variables between the three famine-exposed groups and the non-exposed group to identify potential con-founders. To examine the independent relationship between the famine exposure and the prevalence of T2DM in adulthood, both univariate and multivariate unconditional logistic regression were used. The unexposed group was the reference. Model 1 was an unadjusted model. Model 2 was adjusted for gender, education status, smoking, drinking and body mass index, marital, central obesity, ethnic groups and residence. Within Model 2, adjusted these potential covariates to control bias. In addition, to assess the potential residence-specific effects of famine exposure on T2DM, analyses were also conducted separately in plain area and mountain area. Moreover, to control and reduce the bias related to age differences between famine exposure subgroups, an “age-balanced” method was adopted by combining non-exposed group and adolescence exposed group as the control group. Results are reported as Odds ratios (ORs) with 95% confidence intervals (CIs). *p* values were based on two-sided tests with a cutoff level for statistical significance of 0.05.

## Result

### Characteristics of the famine-exposed and unexposed participants

The basic characteristics of participants exposed to famine during infant, childhood and adolescence stages, and non-exposed participants are presented in Table 1. Among all 3,355 participants, 776 (23.13%) were observed with no exposure to famine; while 490 (14.61%), 1,322 (39.40%) and 767 (22.86%) were observed with famine exposure during infant, childhood and adolescence stages, respectively. 22.35% of participants were diagnosed with T2DM, of which 43.47% were from the childhood famine-exposed group, representing the highest proportion among all subgroups (*p* < 0.001). When compared to the famine-exposed group, participants in the non-exposed group were found to have significantly lower levels of HbA1c, FBG, TG, TC, HDL-C, and LDL-C (*p* < 0.001), as well as reduced incidences of hypertension (*p* < 0.001) and central obesity (*p* < 0.001). In total, 2,346 (69.93%) participants came from the mountain area while 1,009 (30.07%) participants came from the plain area.

**Table 1:** Characteristics of participants according to the Chinese famine exposure.

Variables	Overall	Unexposed	Famine exposure			*p*-value
			Infant	Childhood	Adolescence	
Total, *n* (%)	3,355 (100.00)	776 (23.13)	490 (14.61)	1,322 (39.40)	767 (22.86)	_
Gender						0.019^a^
Male	1,553 (46.29)	325 (41.88)	229 (46.73)	617 (46.67)	382 (49.80)	
Female	1802 (53.71)	451 (58.12)	261 (53.27)	705 (53.33)	385 (50.20)	
Residence						0.016^a^
Plain area	1,009 (30.07)	232 (22.90)	149 (14.77)	365 (36.17)	263 (26.07)	
Mountain area	2,346 (69.93)	544 (23.19)	341 (14.54)	957 (40.79)	504 (21.48)	
Ethnic groups, *n* (%)						0.179^a^
Han	2,447 (72.94)	541 (69.72)	372 (75.92)	963 (72.84)	571 (74.45)	
Hui	862 (25.69)	222 (28.61)	111 (22.65)	345 (26.10)	184 (21.35)	
Others	46 (1.37)	13 (1.68)	7 (1.43)	14 (1.06)	12 (1.56)	
Education, *n* (%)						<0.001^a^
Primary school or below	2,205 (65.72)	352 (45.36)	223 (45.51)	985 (74.51)	645 (84.09)	
Middle school	792 (23.61)	311 (40.08)	177 (36.12)	222 (16.79)	82 (10.69)	
High school or above	358 (10.67)	113 (14.56)	90 (18.37)	115 (8.70)	40 (5.22)	
Marital, *n* (%)						<0.001^a^
Married	2,891 (86.17)	732 (94.33)	463 (94.49)	1,160 (87.75)	536 (69.88)	
Unmarried	464 (13.83)	44 (5.67)	27 (5.51)	162 (12.25)	231 (30.12)	
Smoking, *n* (%)						0.472^a^
No	2,571 (76.63)	364 (74.29)	590 (76.03)	1,027 (77.69)	590 (76.92)	
Yes	784 (23.37)	126 (25.71)	186 (23.97)	295 (22.31)	177 (23.08)	
Drinking, *n* (%)						<0.001^a^
No	2,929 (87.30)	657 (84.66)	418 (85.31)	1,157 (87.52)	697 (90.87)	
Yes	426 (12.70)	119 (15.34)	72 (14.69)	165 (12.48)	70 (9.13)	
BMI (kg/m^2^), Mean ± SD	25.64 ± 3.73	25.88 ± 3.34	25.81 ± 3.30	25.66 ± 3.97	25.24 ± 3.92	0.004^b^
BMI, kg/m^2^, *n* (%)						0.019^a^
<24.0	1,084 (32.31)	226 (29.12)	148 (30.20)	422 (31.92)	288 (37.55)	
24.0 ~ 27.9	1,474 (43.93)	358 (46.13)	229 (46.73)	580 (43.87)	307 (40.03)	
≥28.0	797 (23.76)	192 (24.74)	113 (23.06)	320 (24.21)	172 (22.43)	
WC (cm), Mean ± SD	87.73 ± 10.39	86.43 ± 9.59	87.15 ± 9.45	88.02 ± 9.97	88.93 ± 12.14	<0.001^b^
Central Obesity, cm, *n* (%)						<0.001^a^
Men <90, women <85	1,588 (47.33)	414 (53.35)	244 (49.80)	590 (44.63)	340 (44.33)	
Men 90–94, women 85–89	676 (20.15)	151 (19.46)	107 (21.84)	281 (21.26)	137 (17.86)	
Men ≥95, women≥90	1,091 (32.52)	211 (27.19)	139 (28.37)	451 (34.11)	290 (37.81)	
Diabetes, *n* (%)	750 (22.35)	129 (17.20)	90 (12.00)	326 (43.47)	205 (27.33)	<0.001^a^
HbA1c(%), Mean ± SD	5.93 ± 0.95	5.83 ± 0.91	5.86 ± 0.86	5.98 ± 0.97	5.97 ± 0.99	<0.001^b^
FBG (mmol/l), Mean ± SD	6.07 ± 1.80	5.86 ± 1.79	5.90 ± 1.44	6.16 ± 1.83	6.22 ± 1.94	<0.001^b^
TG (mmol/l), Mean ± SD	1.58 ± 1.28	1.64 ± 1.22	1.71 ± 1.69	1.58 ± 1.31	1.43 ± 0.94	<0.001^b^
TC (mmol/l), Mean ± SD	4.55 ± 1.03	4.65 ± 1.01	4.60 ± 0.99	4.56 ± 1.02	4.38 ± 1.06	<0.001^b^
HDL-C (mmol/l), Mean ± SD	1.25 ± 0.28	1.27 ± 0.28	1.27 ± 0.27	1.25 ± 0.27	1.22 ± 0.28	<0.001^b^
LDL-C (mmol/l), Mean ± SD	2.79 ± 0.81	2.87 ± 0.79	2.79 ± 0.77	2.79 ± 0.81	2.70 ± 0.84	<0.001^b^

BMI, body mass index; HbA1c, glycated hemoglobin; FBG, fasting blood glucose; TG, triglycerides; TC, total cholesterol; WC, waist central; HDL-C, high-density lipoprotein cholesterol; LDL-C, low-density lipoprotein cholesterol.

a *p* values were obtained from chi-square test.

b *p* values were obtained from ANOVA.

### Association of early-life famine exposure and T2DM across different areas

**Figure 2:**
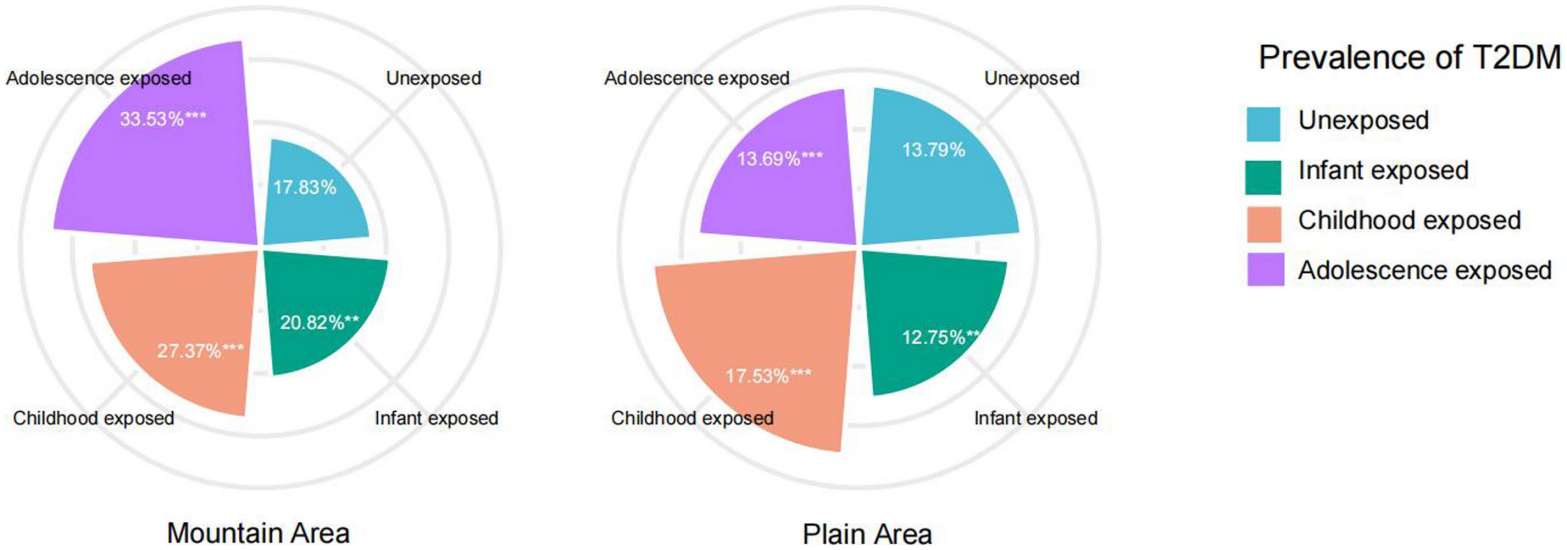
Prevalence of type 2 diabetes mellitus in mountain area and plain area. ***p < 0.001, **p < 0.05.

Figure 2 compares the prevalence of T2DM between mountainous and plain areas. In the plain area, the prevalence of T2DM were observed as 12.75, 17.53, and 13.69% in the infant famine-exposed group, childhood famine-exposed group and adolescence famine-exposed group, respectively. In the mountain area, the prevalence of T2DM were observed as 20.82, 27.37 and 33.53% in the infant famine-exposed group, childhood famine-exposed group and adolescence famine-exposed group, respectively. Participants exposed to famine during childhood and adolescence and from mountainous areas showed a significantly higher prevalence of T2DM in adulthood than those from the plain areas (*p* < 0.001). No statistical differences were observed for the infant famine-exposed group between two areas.

### Odds ratios of T2DM across various famine-exposed stages

**Figure 3:**
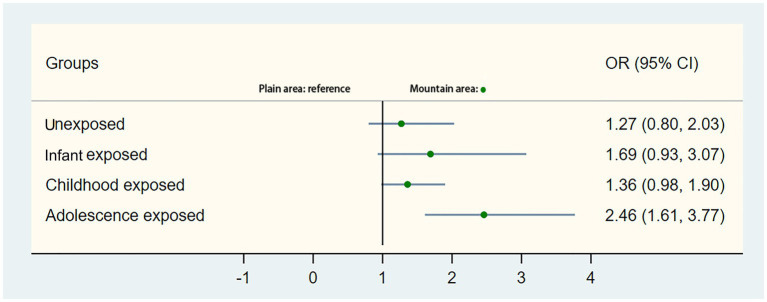
Stratification analysis of associations of mountain area and plain area with the risk of type 2 diabetes mellitus. Presented were multivariable-adjusted generalized linear models with adjustment for covariates. The circle in the middle represents the odds ratio of the risk estimation, and the bar represents its 95% CI.

Table 2 presents the association between the occurrence of T2DM and different stages of famine exposure. After adjusting for gender, educational status, BMI, smoking, alcohol drinking, ethnic groups, marital status, central obesity and residence, the overall odds ratios (ORs) were 1.65 (95%CI: 1.30–2.09) for the childhood famine-exposed group and 1.89 (95%CI: 1.44–2.49) for the adolescence famine-exposed group. Significantly differences in ORs were observed for the childhood famine-exposed and the adolescence famine-exposed groups in the mountain area, which were 1.76 (95%CI: 1.34–2.31) and 2.35 (95%CI: 1.72–3.22) respectively. No statistical differences in ORs were observed for these two groups in the plain area. Further combined non-exposed group and adolescence exposed group as the control group, no statistical differences in ORs were observed for infant famine-exposed and childhood famine-exposed groups (Supplementary Table S1). As presented in Supplementary Table S2, significant statistical differences were also observed between mountain and plain areas (OR 1.64, 95%CI 1.33–2.02). The Figure 3 also showed that the adolescence stage exposed famine will increase the risk of T2DM in the mountain area (OR 2.46, 95% CI 1.61, 3.77).

**Table 2:** The association between early life famine exposure and the risk of type 2 diabetes.

Variables	Unexposed	Famine exposure					
		Infant	*p*-value	Childhood	*p*-value	Adolescence	*p*-value
**Overall**							
Model 1	1.00 (reference)	1.13 (0.84, 1.52)	0.424	1.64 (1.31, 2.06)	<0.001	1.83 (1.43, 2.34)	<0.001
Model 2	1.00 (reference)	1.13 (0.83, 1.53)	0.440	1.65 (1.30, 2.09)	<0.001	1.89 (1.44, 2.49)	<0.001
**Mountain area**							
Model 1	1.00 (reference)	1.21 (0.86, 1.70)	0.270	1.74 (1.34, 2.26)	<0.001	2.32 (1.74, 3.09)	<0.001
Model 2	1.00 (reference)	1.23 (0.87, 1.73)	0.249	1.76 (1.34, 2.31)	<0.001	2.35 (1.72, 3.22)	<0.001
**Plain area**							
Model 1	1.00 (reference)	0.91 (0.50, 1.68)	0.771	1.33 (0.84, 2.11)	0.226	0.99 (0.59, 1.66)	0.973
Model 2	1.00 (reference)	0.87 (0.46, 1.63)	0.651	1.35 (0.80, 2.67)	0.256	0.95 (0.52, 1.73)	0.870

Model 1 Unadjusted; Model 2 adjusted for gender, education status, smoking, drinking and body mass index, marital, central obesity, ethnic groups, and residence (only for overall).

### Stratified analysis of the association between famine exposure and T2DM

The comparisons of ORs for the occurrence of T2DM among participants with and without exposure to famine are showed in Table 3. Statistically interaction effects were observed in terms of residence, gender (*p* < 0.05). However, no significant interaction effects were observed between the BMI, central obesity, education status, marital status, smoking and drinking subgroups.

**Table 3:** Stratified analysis of early life famine exposure and the risk of type 2 diabetes.

Variables	Unexposed	Famine exposure			*p*_interaction_
		Infant	Childhood	Adolescence	
Residence, *n* (%)					<0.05
Plain area	1.00 (ref.)	0.85 (0.43, 1.69)	1.30 (0.53, 3.17)	0.88 (0.21, 3.72)	
Mountain area	1.00 (ref.)	1.02 (0.70, 1.49)	1.07 (0.64, 1.79)	0.93 (0.39, 2.20)	
Gender, *n* (%)					<0.05
Male	1.00 (ref.)	1.00 (0.62, 1.63)	1.42 (0.75, 2.67)	1.47 (0.51, 4.23)	
Female	1.00 (ref.)	0.95 (0.60, 1.49)	0.89 (0.48, 1.66)	0.61 (0.21, 1.72)	
BMI, kg/m^2^, *n* (%)					0.686
<24.0	1.00 (ref.)	1.15 (0.52, 2.54)	2.52 (0.98, 6.50)	2.29 (0.48, 11.06)	
24.0 ~ 27.9	1.00 (ref.)	0.90 (0.56, 1.44)	0.76 (0.39, 1.48)	0.43 (0.14, 1.32)	
≥28.0	1.00 (ref.)	0.95 (0.53, 1.73)	1.00 (0.46, 2.20)	1.36 (0.38, 4.91)	
Central Obesity, cm, *n* (%)					0.507
Men <90, women <85	1.00 (ref.)	1.53 (0.89, 2.61)	1.99 (0.94, 4.23)	1.81 (0.50, 6.56)	
Men 90–94, women 85–89	1.00 (ref.)	0.56 (0.27, 1.13)	0.69 (0.27, 1.79)	0.50 (0.10, 2.48)	
Men ≥95, women≥90	1.00 (ref.)	0.90 (0.53, 1.54)	0.92 (0.46, 1.82)	0.76 (0.25, 2.32)	
Education, *n* (%)					0.171
Primary school or below	1.00 (ref.)	0.98 (0.60, 1.61)	1.14 (0.63, 2.06)	1.11 (0.43, 2.78)	
Middle school	1.00 (ref.)	1.09 (0.63, 1.89)	1.01 (0.42, 2.44)	0.56 (0.11, 2.91)	
High school or above	1.00 (ref.)	0.54 (0.22, 1.32)	0.80 (0.21, 3.08)	0.33 (0.02, 4.62)	
Marital, *n* (%)					0.905
Married	1.00 (ref.)	0.95 (0.68, 1.35)	1.04 (0.65, 1.68)	0.98 (0.44, 2.20)	
Unmarried	1.00 (ref.)	2.19 (0.49, 9.77)	4.41 (0.96, 20.26)	2.70 (0.32, 23.04)	
Smoking, *n* (%)					0.087
No	1.00 (ref.)	1.14 (0.57, 2.25)	1.09 (0.43, 2.75)	0.84 (0.17, 4.15)	
Yes	1.00 (ref.)	0.94 (0.64, 1.37)	1.15 (0.69, 1.92)	0.98 (0.42, 2.29)	
Drinking, *n* (%)					0.436
No	1.00 (ref.)	1.08 (0.76, 1.54)	1.12 (0.70, 1.81)	0.92 (0.42, 2.02)	
Yes	1.00 (ref.)	0.51 (0.18, 1.40)	1.39 (0.37, 5.20)	1.34 (0.11, 15.69)	

BMI, body mass index; Multivariable model was adjusted for age, gender, education status, residence, smoking, drinking and body mass index, marital, central obesity, and ethnic groups.

## Discussion

Our study found that when accounting for the age difference between the comparison groups, exposure to famine during early life stages did not demonstrate a correlation with an increased risk of T2DM in adulthood. Uncontrolled age discrepancies between famine births and post famine births is potent to exaggerate the perceived association between early-life exposure to famine and the risk of developing T2DM in later years (22, 23).

While our analysis of combined data of individuals from both mountainous and plain regions did not reveal an association between early-life exposure to famine and risk of T2DM in adulthood, a nuanced examination focusing exclusively on individuals from mountainous regions (those at lower SES) uncovered that exposure to famine during adolescence can increase the risk of developing T2DM later in life (OR 2.46 95% CI:1.61, 3.77). One possible explanation is that the low SES exposes populations in the NHAR mountains to more potential risk factors of T2DM than in the plains where SES is higher. Some prospective population-based studies have also accessed this correlation. In England it was shown that the incidence of T2DM is increased in lower SES even with the adjustment for regular risk factors for T2DM (24). In the USA, based on the data from Women Heath Study, researcher demonstrated an inverse relationship between increasing education and income to diabetes incidence (25). In our study, the southern mountainous areas include Xiji, Haiyuan, and Yanchi counties, also known as XiHaiGu. This region has been recognized as one of the most barren areas in China since ancient times (18). It was once categorized as one of the national contiguous destitute areas and was evaluated by the United Nations Food and Agriculture Organization (FAO) in 1972 as “one of the most unsuitable areas for living” (18). For a long time, this region has faced a “PPE (poor, people, and environment) vicious circle,” presenting a persistent contradiction between population, resources, environment, and socioeconomic development. Furthermore, several studies found that in T2DM, relative insulin deficiency owing to β-cell dysfunction is a key factor for the development of disease (26–28) that often coexists with insulin resistance. Food restriction in the early life might decrease the beta cell mass, and possibly a less favorable gut microbiota may also be related to increase the T2DM rates (29, 30). Previous studies have found a clear association between the socioeconomic gradient in T2DM and the utilization of healthcare resources (31, 32). Miller found that people living with T2DM may demonstrate lower level of adherence to healthcare interventions once they perceive that these interventions these interventions impose a financial burden on their disposable income (33). Reduced consistency in using medications and adhering to recommended guidelines can increase the risk of diabetes-related complications. On the other hand, people who living in the underdeveloped mountain or low SES areas have even less opportunities to access to healthcare services (32), limiting their engagement with the early screening of T2DM or chronic disease screenings, particularly as digital healthcare has not been widespread in these areas (34). There is also evidence that economic conditions in early life can have lasting influences throughout the lifecycle. For example, using historical data from the Netherlands (19), compare individuals born during the economic boom of 1872 to 1876 with individuals born during the recession of 1877 to 1881, they founded that people born during economically prosperous years had life expectancies that were about 1.6 years longer than those born during the economic downturn.

In fact, maternal nutrition may play a key role in their offspring’s risk of non-communicable diseases (NCDs) later in life. This is a profoundly important relationship, particularly in low and lower-middle income countries (LLMICs) who face the double burden of malnutrition, and 80% of the global NCD burden (35). Furthermore, participants in our study who born in the mountain area may have lower birth weight, which may related to the nutritional status of their mother during gestation. They tend to present a higher body fat mass and diabetes risk (36). Previous study also observed that low birth weight is associated with increased rates of obesity, insulin resistance and T2DM (37). A study suggested that low fat deposition leading to thinness at birth and during infancy results in fat acquisition during childhood, with a subsequent increase in the risk of developing T2DM (38). The finding was consistent with our result. After adjusting the covariates, the childhood exposure group had a high risk of T2DM [OR:1.76 95% CI(1.34, 2.31)]. Overall, our results suggest that people born with a low weight should be advised regarding the risks of developing T2DM.

We also observed significant interaction effects in gender in our study that females exposed to famine during the early-life stage had a higher risk of T2DM than males. We speculate that this could be attributed to the relatively poorer health status of Chinese women compared to men over the last century, as reported by Zhao et al. (39). According to the concept of “mortality selection” (40), which was defined as the tendency for individuals with higher levels of exposure to disadvantageous characteristics to be more likely to experience die, female participants in this study appear to have been exposed to a greater degree of disadvantage during the famine.

The study includes several key strengths. It examines the effect of a historical natural event, the Great Chinese Famine, on people’s health status. The unique topography of NHAR provided a natural classification of famine severity in this region. This study is also based on high-quality data, which was derived from a representative sample across nine counties in NHAR. However, this study also has some limitations. The primary challenge is the vague timeline of the Great Chinese Famine, making it difficult to categorize participants accurately based on their birth dates. Potential misclassification of subgroups may presented. Additionally, the lack of follow-up data may limit our ability to account for the direct effects of time intervals on the study’s findings.

## Conclusion

No strong evidence demonstrates that exposure to famine during the early life stage can increase the risk of developing T2DM in adulthood. However, populations with lower SES are likely to be exposed to more risk factors for T2DM. It is recommended that future policies and interventions aimed at chronic disease management should pay more attention to vulnerable populations.

## Data availability statement

The raw data supporting the conclusions of this article will be made available by the authors, without undue reservation.

## Ethics statement

This study was conducted according to the guidelines laid down in the Declaration of Helsinki and all procedures involving human subjects were approved by the Institutional Review Board of the People's Hospital of Ningxia Hui Autonomous (No. 2020-YC-002). Verbal consent was witnessed and formally recorded. Informed consent was confirmed by the IRB the People's Hospital of Ningxia Hui Autonomous.

## Author contributions

HS: Conceptualization, Formal analysis, Methodology, Visualization, Writing – original draft, Writing – review & editing. DY: Conceptualization, Formal analysis, Methodology, Visualization, Writing – original draft, Writing – review & editing. LM: Data curation, Formal analysis, Writing – review & editing. YC: Data curation, Formal analysis, Writing – review & editing. YL: Investigation, Writing – review & editing. JM: Investigation, Writing – review & editing. HT: Investigation, Writing – review & editing. CS: Conceptualization, Funding acquisition, Methodology, Project administration, Supervision, Validation, Visualization, Writing – review & editing, Formal analysis.
