# Supplementary material for: Early life malnutrition and risk of T2DM adulthood: evidence from the lower socioeconomic status of northwest Chinese population
Source: Front Nutr. 2024 Jun 27;11:1379725. doi: 10.3389/fnut.2024.1379725 (PMC11236714; doi:10.3389/fnut.2024.1379725)
Supplement: Supplementary file 1 [file Data_Sheet_1.docx]

Supplementary Material

# Supplementary Figures and Tables

## Supplementary Figures

**Supplementary Figure 1** Flow diagram of study participants


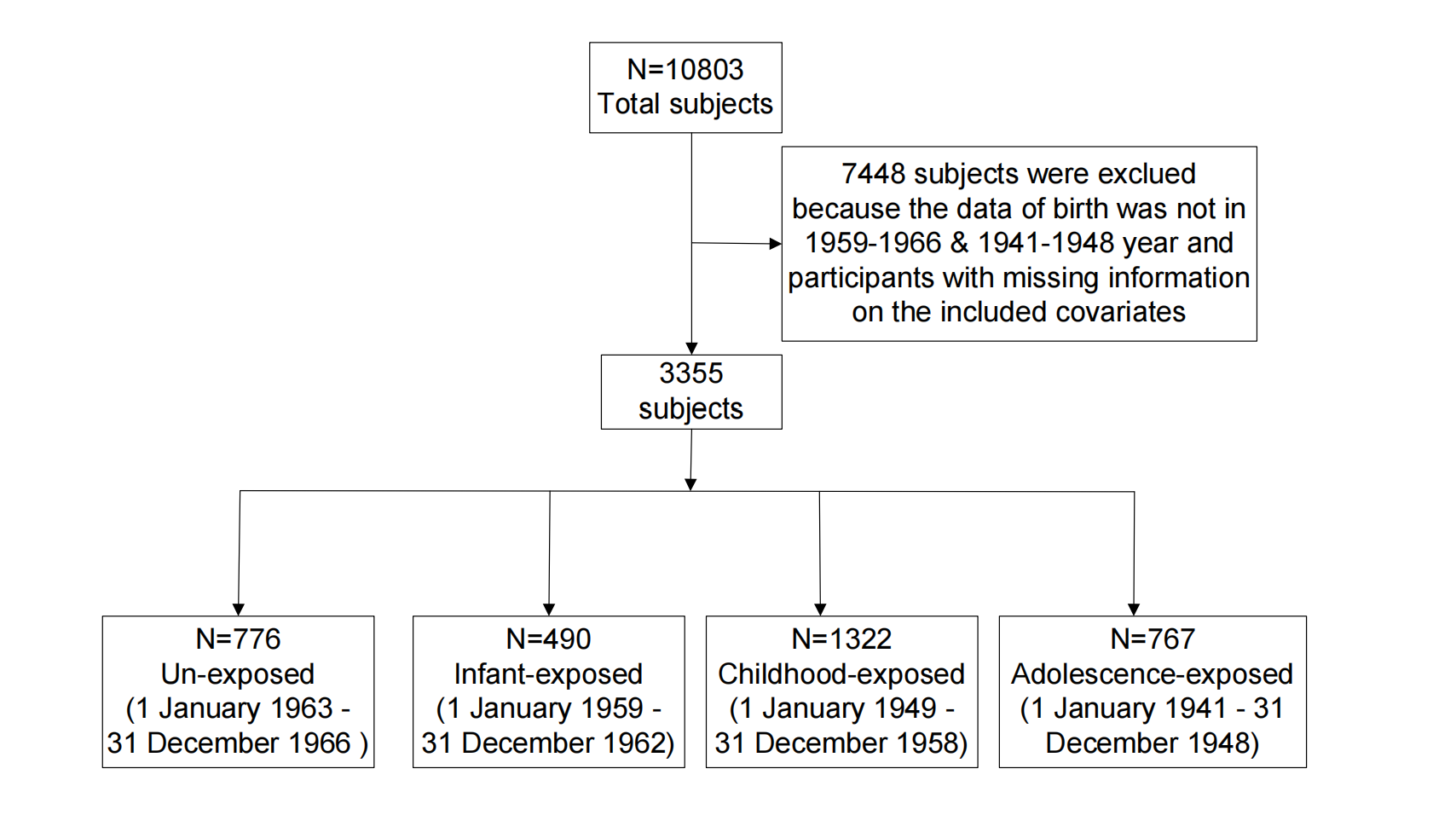


## Supplementary Tables

| Supplementary Table 1 ORs (95% CIs) for type 2 diabetes according to famine exposure in early life compared with age-balanced controls. | | | | | |
| --- | --- | --- | --- | --- | --- |
| Variables | Age-balanced control | Infant exposed | *P* value | Childhood-exposed | *P* value |
| Overall | | | | | |
| Model 1 | 1.00 (reference) | 0.81 (0.63,1.05) | 0.120 | 1.18 (1.00,1.41) | 0.056 |
| Model 2 | 1.00 (reference) | 0.79 (0.61,1.04) | 0.188 | 1.16 (0.97,1.39) | 0.093 |
| Mountain area | | | | | |
| Model 1 | 1.00 (reference) | 0.77 (0.57,1.04) | 0.088 | 1.11 (0.91,1.35) | 0.311 |
| Model 2 | 1.00 (reference) | 0.78 (0.58,1.06) | 0.208 | 1.12 (0.91,1.37) | 0.277 |
| Plain area | | | | | |
| Model 1 | 1.00 (reference) | 0.92 (0.53 ,1.58) | 0.758 | 1.34 (0.92,1.94) | 0.128 |
| Model 2 | 1.00 (reference) | 0.80 (0.46,1.41) | 0.673 | 1.33 (0.90,1.96) | 0.104 |
| Model 1 Unadjusted; Model 2 adjusted for gender, education status, smoking, drinking and body mass index, marital, central obesity,ethnic grops and residence(only for overall) | | | | | |

| Supplementary Table 2 Risk factors for diabetes in 3355 individuals from Ningxia Hui Autonomous Region, China | | |
| --- | --- | --- |
| Variables | Model 1 | Model 2 |
| Gender |  |  |
| Female | reference | reference |
| Male | 0.97 (0.82,1.14) | 1.12 (0.90,1.38) |
| Residence |  |  |
| Plain area | reference | reference |
| Mountain area | 1.95 (1.60,2.37) | 1.64 (1.33,2.02) |
| Education, n (%) |  |  |
| Primary school or below | reference | reference |
| Middle school | 1.01 (0.83,1.23) | 1.12 (0.90,1.39) |
| High school or above | 1.13 (0.87,1.47) | 1.32 (0.99,1.76) |
| Marital, n (%) |  |  |
| Unmarried | reference | reference |
| Married | 0.82 (0.65,1.03) | 0.91 (0.71,1.16) |
| Smoking, n (%) |  |  |
| No | reference | reference |
| Yes | 0.77 (0.64,0.95) | 0.84 (0.65,1.08) |
| Dringking, n (%) |  |  |
| No | reference | reference |
| Yes | 0.83 (0.65,1.08) | 0.87 (0.65,1.16) |
| BMI, kg/m2, n (%) |  |  |
| <24.0 | reference | reference |
| 24.0~27.9 | 1.98 (1.61,2.45) | 1.60 (1.27,2.01) |
| ≥28.0 | 2.96 (2.36,3.73) | 1.89 (1.42,2.51) |
| Central Obesity,cm, n (%) |  |  |
| Men <90, women <85 | reference | reference |
| Men 90-94, women 85-89 | 1.86 (1.49,2.33) | 1.45 (1.14,1.85) |
| Men ≥95, women≥90 | 2.78 (2.30,3.35) | 1.84 (1.46,2.33) |
| Model 1 Unadjusted; Model 2 adjusted for gender, education status, body mass index, smoking, drinking and residence,martail, central obesity | | |
